# The physiological measurement and evaluation of empathy of video content

**DOI:** 10.1038/s41598-023-47288-1

**Published:** 2023-11-18

**Authors:** Ayoung Cho, Sung Park, Hyunwoo Lee, Mincheol Whang

**Affiliations:** 1 Department of Emotion Engineering, Sangmyung University, Seoul, South Korea; 2 Department of Human-Centered Artificial Intelligence, Sangmyung University, Seoul, South Korea

**Keywords:** Physiology, Psychology

## Abstract

The COVID-19 pandemic has led to a surge in video content consumption, but measuring viewers' empathy towards the content has been limited to subjective evaluations or attached physiological apparatus. In this study, we introduced a novel non-contact physiological method for measuring empathy towards video content by assessing the synchronization of facial micromovements between the subject and object (i.e., person) within the media. We recorded facial micromovements and heart rate variability (HRV) remotely using a camera while 62 subjects watched one video each, designed and validated to elicit one of four two-dimensional emotions: pleasant-aroused, pleasant-relaxed, unpleasant-aroused, and unpleasant-relaxed. We also collected the subjects' self-assessed emotions and empathy using a questionnaire. The results confirmed that the stimuli effectively induced the intended arousal in the subjects, as evidenced by both self-reported emotions and HRV responses that suggested higher arousal was associated with stronger activity in the sympathetic nervous system. A closer examination of HRV indicators, such as SDNN and Total Power values, showed an amplification during the unpleasant state. We interpret this as the body's dynamic response to stressors, underlining the autonomic nervous system's proactive role in responding to such stimuli. In a broader context, our results emphasized that while subjects showcased augmented empathy during aroused conditions, the introduction of stressors, represented by unpleasant content, led to a dampening of this empathetic response. This findings demonstrate the potential of non-contact physiological methods for measuring empathy toward video content.

## Introduction

The consumption of video-based content has risen exponentially amid the pandemic. Specifically, the viewership of video-on-demand (VOD) and real-time television has significantly increased. (https://www.forbes.com/sites/falonfatemi/2021/02/01/how-the-pandemic-has-changed-video-content-and-consumption/?sh=3eebda286ec0) While offline content (i.e., theater-based shows) has completely halted digital content, any content in the form of digital data, whether streamed or broadcast, has massively been consumed. Particularly, video content, including TV shows, movies, ads, news, and music videos, and user-created content (UCC) with the popularity of more concise and impactful “shorts” has proliferated, especially among the younger generation.

The success of such content largely depends on whether the consumer empathizes with it. Specifically, whether a user with a varying degree understands the thoughts, behavior, and emotions associated with the character and story becomes an essential criterion for successful content^1^. As a content creator, it is interesting to measure the user’s empathy when viewing the content. Traditionally, measuring empathy has been limited to content-wise advertisements and subjective evaluation methods. Numerous scale items measure empathy involving commercials, including the scales to measure emotional responses by Escalas and Stern^2–5^. However, such measures require the subject to respond to scales after viewing the target content. As emotion fluctuates as a function of time when viewing video content, more real-time and direct measures are required, such as physiological measures, to measure empathy accurately.

Empathic mirroring responses between a dyadic pair encompass both explicit and implicit physiological synchronization^6–8^. The overt reactions involve synchronized body movements, gestures, and facial expressions^9,10^. For instance, increased head motion mirroring was noted in situations where listeners empathized with a speaker^11^. Such mirroring has also been observed in therapeutic environments^12,13^.

These observed behaviors are the result of an implicit physiological reactions^14^. Physiological synchronization can be observed through skin conductance^15,16^, electroencephalography (EEG)^17,18^, and electrocardiography (ECG)^19–22^. Notably, recent studies, Maffei et al.^23^ measured EEG gamma band activity in women with varying empathy levels, noting that those with high empathy displayed greater arousal and gamma activity, especially when viewing compassion clips. Lettieri et al.^24^ identified spatial gradients in the right temporo-parietal brain region encoding the complexity and intensity of emotions, introducing the concept of 'emotionotopy.' Nummenmaa et al.^25^ revealed that during movie viewing, negative valence increased synchronized brain activity in emotion-processing networks, suggesting that emotions might enhance social interactions. The perceived level of empathy has been shown to correlate with synchronization metrics, such as those observed in skin conductance measures^26,27^.

Cardiovascular metrics, reflecting both affective and attentional states, have also emerged as promising tools in empathy research^28^. While historically understudied compared to EEG and skin conductance^29^, recent studies, such as those by^30,31^, have highlighted the potential of cardiovascular synchrony as an empathy indicator. However, Maffei et al.’s findings^23^ on experimental movies further extend the understanding of emotional elicitation, underscoring the need to explore varied stimuli types when assessing physiological responses.

Measuring synchronization between cardiovascular activities of viewers and those portrayed in visual content remains nascent, primarily due to the challenges of employing sensory ECG apparatus. However, advancements like remote photoplethysmography (rPPG) and rBCG provide avenues to address these limitations. The rPPG detects changes in blood volume remotely without direct contact between the photosensor and skin^32^. The rPPG uses band-pass filters to eliminate motion components in images^33^ but has a lower effect on cardiovascular activities that include the motion itself, referred to as ballistocardiography motion^34^.

rBCG is a technique that utilizes remote camera-based vision analysis to measure ballistocardiographic head movements. BCG motion causes minute vibrations, known as micromovement, which are transmitted to the face via the carotid artery. Micromovement refers to the subtle facial movements that are not easily discernible to the human eye, driven by regular vibrations from the heart. Filtering the frequency that corresponds to the regular heart rate band from frontal facial video capture enables us to capture micromovement^35–38^. It is important to note that such micromovement is distinct from micro-facial expressions, which are spontaneous and involuntary facial movements that occur when a person experiences an emotion^39,40^. We chose to use rBCG because it has been shown to be an effective method for analyzing micromovements in experimental settings where subjects watch videos with minimal head movement, as demonstrated by Moco et al.^34^.

In light of the insights from^23–25, 41^, our study aims to leverage rBCG to discern if facial micromovements can signify empathetic reactions to video content. Given the intricate relationship between stimulus types and neural correlates, we hypothesize that our results will offer novel perspectives on empathetic viewer responses to video content. Specifically, we created videos to induce varying arousal and valence emotions in subjects. We collected self-assessed emotions and empathy measures from participants after video viewing and physiological responses (i.e., HRV) during viewing. We also calculated cross-entropy measures using facial micromovement signals through rBCG, and compared these measures to self-assessed measures. This study is the first to use rBCG to assess viewer empathy toward video content.This paper outlines our experimental procedures, stimuli, methods, and our consequent findings, culminating in a discussion on their implications.

## Methods

### Subjects

A power analysis was performed using G*Power^42^ to determine the number of subjects required for valid inferences. At least 54 subjects were required to perform the F-test with four repetitions, 95% statistical power and 0.4 effect size. We recruited 62 university students (29 males) as subjects with an average age of 22 ± 2.0. The subjects had no medical history related to the visual system, autonomic nervous system, or facial muscles. All subjects had an uncorrected or corrected visual acuity of 0.6 or were able to wear soft and hard lenses. Written informed consent was obtained from all subjects prior to the experiment. This study was conducted according to the recommendations of the Institutional Review Board of Sangmyung University, Seoul, Korea (BE2018-35).

### Experiment design and materials

This study aims to identify the difference in micromovement synchronization and understand its physiological implications through heart rate variability (HRV) when subjects view stimuli that elicit varying emotions. We adopted Russell’s two-dimensional emotional model^43^, which represents various emotions in two dimensions: valence and arousal. The experiment was a two (valence: pleasant and unpleasant) by two (arousal: aroused and relaxed) within-subjects design. We measured and analyzed subjects’ facial and HRV data as dependent measures.

**Figure 1:**
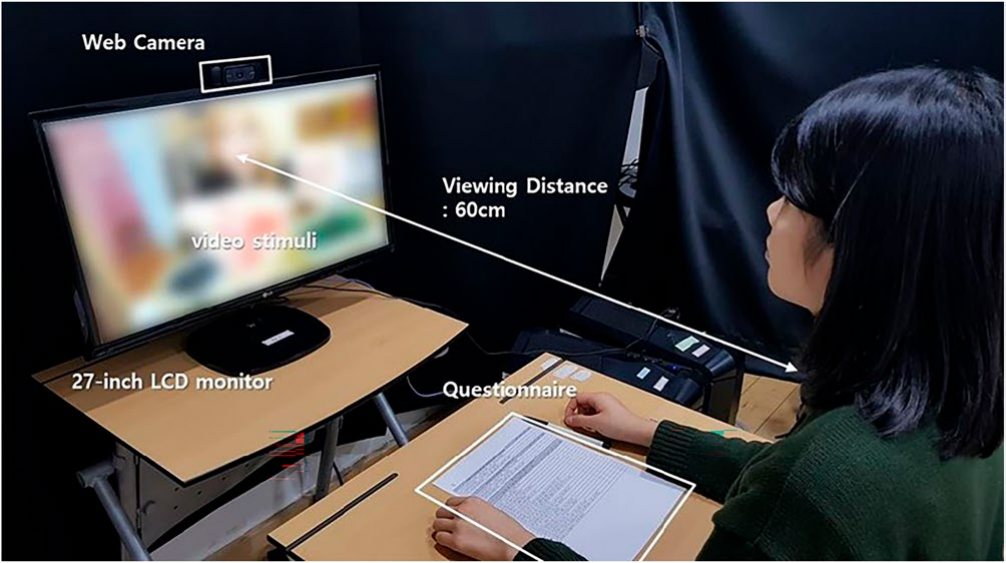
Experimental environment.

Figure 1 shows the experimental environment of this study. The subjects were seated 0.6 m from a 27-inch LCD monitor. The facial data of the subjects were acquired using a webcam installed on the monitor. A Logitech c920 webcam (Logitech, Lausanne, Switzerland) was used to obtain image data with a resolution of 1920 × 1080 pixels at 30 frames per second. The subjects were allowed to freely express their facial expressions and move their heads while viewing the video stimuli. Electrocardiogram (ECG) data were acquired to analyze the activation level of the autonomic nervous system (ANS) when subjects viewed visual stimuli. A Biopac system (Biopac, Goleta, CA, USA) with a frequency of 500 Hz was used. A self-assessed empathy questionnaire was placed in front of the subjects to respond to the subjective evaluation immediately after watching the video stimuli.

**Figure 2:**
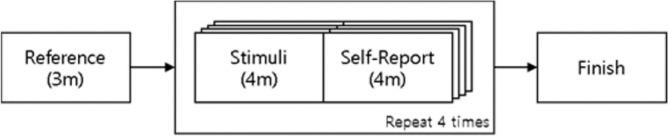
Experimental procedure.

Figure 2 outlines the experimental procedure for comparing empathy and the corresponding micro-movement. Before the stimuli, all subjects stared at the blank screen for three minutes (i.e., the reference task) to stabilize their physiological state without the stimulus. The subjects then viewed each video intended to elicit one of the following two-dimensional emotions: pleasant-aroused, pleasant-relaxed, unpleasant-aroused, and unpleasant-relaxed. They viewed the videos for four minutes in random order. While the subjects viewed the video, their facial data were obtained at 30 fps and 1920 × 1080 pixels using the webcam, in addition to the HRV measures using the ECG apparatus. After watching the video, the subjects responded to a self-assessed empathy questionnaire on a five-point Likert scale for four minutes. The design of the questionnaire is explained in "Empathy score" section.

#### Video stimuli

Media content appealing to emotions induces higher empathy than appealing to reason, and storytelling techniques lead to higher empathy than listing facts or statistical evidence^2,44^. We collected YouTube video clips with characters conveying emotion and content through storytelling to induce empathy. Fifteen researchers screened the clips through a focused group discussion and selected a candidate clip for all four quadrants in Russell’s two-dimensional emotion model^43^: low valence and high arousal (unpleasant-aroused), high in both valence and arousal (pleasant-aroused), high in valence and low in arousal (pleasant-relaxed), and low in both valence and arousal (unpleasant-relaxed).

We conducted a stimuli manipulation check with 227 subjects who viewed the candidate clips and scored the arousal (1: very relaxed, 2: relaxed, 3: neutral, 4: aroused, 5: very aroused), valence (1: very unpleasant, 2: unpleasant, 3: neutral, 4: pleasant, 5: very pleasant), and empathy levels (1: strongly disagree, 2: disagree, 3: neutral, 4: agree, 5: strongly agree) of the four candidate clips on a five-point Likert scale. Subjects’ ages ranged from 20 to 30 with 106 male (47%) and 121 (53%) female subjects.

**Figure 3:**
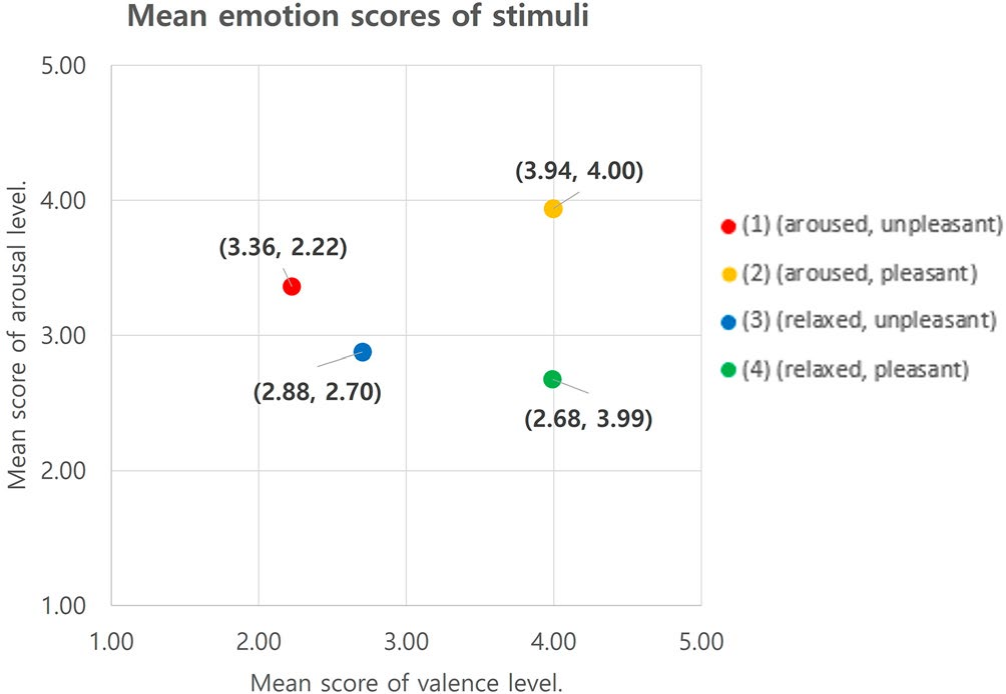
Video stimulus for each quadrant on a two-dimensional model.

**Figure 4:**
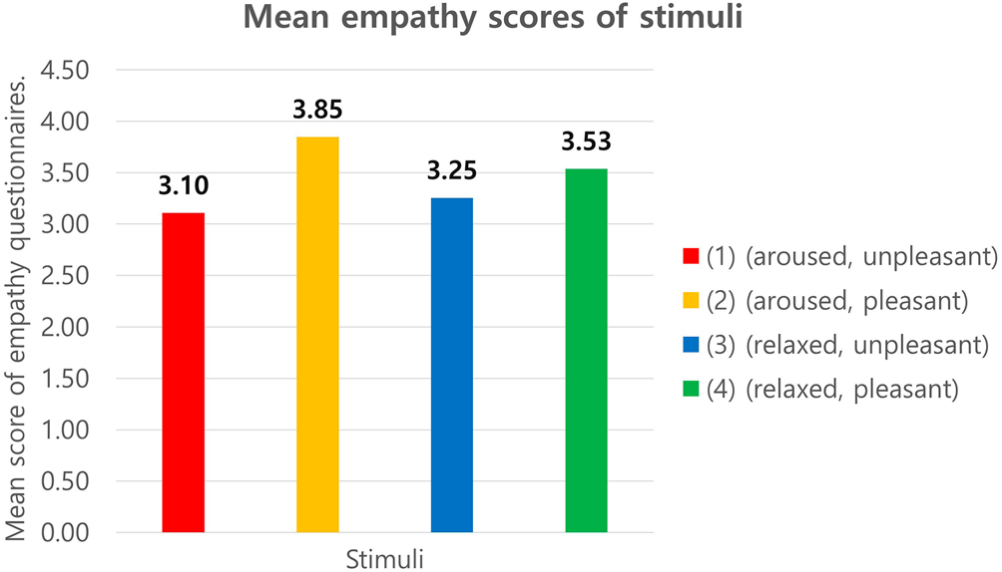
Signal processing of the micromovements.

After verifying the average arousal and valence scores from all subjects, it was confirmed that all four clips elicited the anticipated levels of arousal and valence within the expected quadrant of the two-dimensional model. For example, as shown in Fig. 3, in clip 1 (unpleasant-aroused), the arousal average score surpassed 3, while the valence average was below 3, as shown in Fig. 3. Each of the four candidates evoked empathy, with their average scores going beyond 3, as highlighted in Fig. 4.

**Figure 5:**
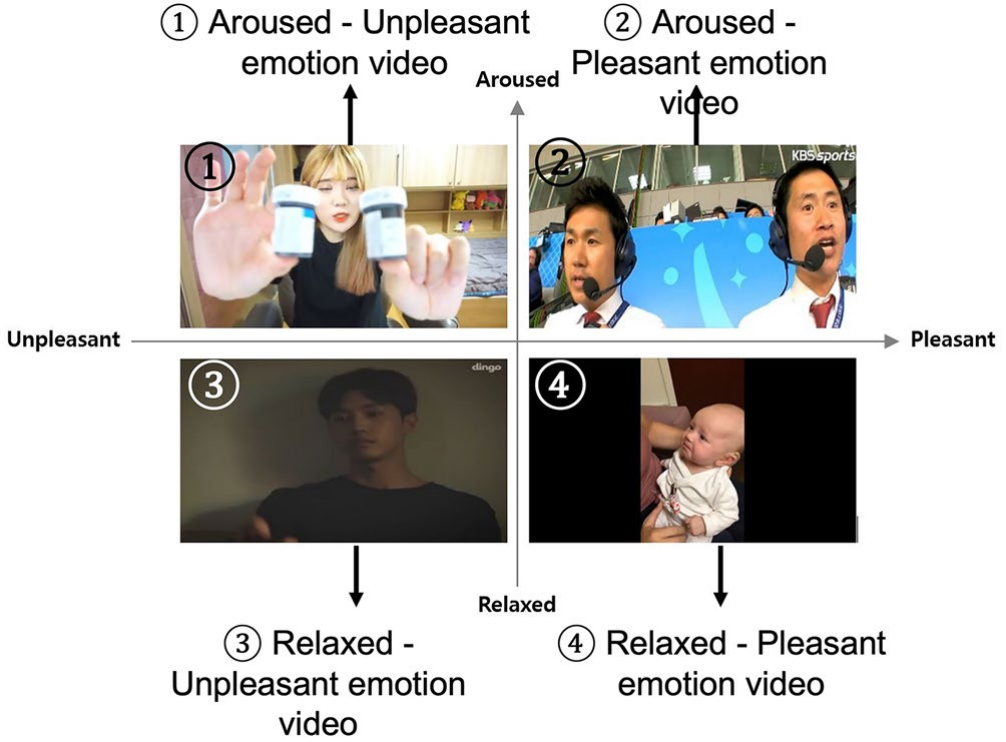
Mean emotion scores of stimuli.

Figure 5 shows the clips selected for the main experiment. The following is a brief description of each video clip. (1) (aroused, unpleasant) A YouTuber introduces and applies blue sauce to her noodles and eats them. (2) (aroused, pleasant) Two sportscasters shout and cheer excitingly at a goal during a soccer game. (3) (relaxed, unpleasant) The actor appears melancholic and sad, blaming himself after the breakup. (4) (relaxed, pleasant) The mother whispers calmly to the baby, and the scene looks serene.

#### Empathy score

There are prominent questionnaires that measure interpersonal empathy, such as the Empathy Quotient (EQ)^45^ or the Interpersonal Reaction Index (IRI)^46,47^. Ad response empathy (ARE) scales have been developed for consumer empathy in commercial advertisements^2^. In addition, empathy for media content has been classified and verified into three groups: cognitive, identification, and affective empathy^48^. Similarly, Rhee et al.^49^ developed and verified an empathy questionnaire for social media video content.

Empathy is a multi-dimensional concept that encompasses our ability to understand, feel, and respond to the emotions and experiences of others (for an extensive review of empathy as a concept, see^50^. While a universally accepted definition remains elusive, researchers often divide empathy into three categories: affective empathy, cognitive empathy, and identification empathy. Affective empathy generally connotes the observer’s visceral reaction to the target’s affective state^51–53^. In contrast, cognitive empathy entails adopting another's viewpoint, deducing their emotions, thoughts, and traits^54–57^. Identification empathy is the profound bond felt when one's identification with another's experience is so intense it feels personally experienced^58,59^. We utilized the empathy questionnaire formulated by Yoo and Whang^60^ due to its comprehensive coverage of these empathy facets. This questionnaire contains 19 items, detailed in Table 1, with a factor loading above 0.4 and a Cronbach’s alpha greater than 0.8.

**Table 1:** Questionnaire of self-assessed empathy on video content.

Questionnaire		Component of empathy
1	I understood the video’s portrayed emotion	Cognitive empathy
2	I understood the characters’ needs	Cognitive empathy
3	I understood how the characters were feeling	Cognitive empathy
4	I felt as if the events in the video could happen to me	Cognitive empathy
5	I understood the situation of the video	Cognitive empathy
6	I understood the characters’ motivation for their actions	Cognitive empathy
7	People like myself can sympathize with the video	Identification
8	I empathized with the message of the video	Identification
9	I felt as if the character’s feelings were similar to my past feelings	Identification
10	I felt as if the character’s needs were similar to my past needs	Identification
11	The events in the video were similar to my experience	Identification
12	I felt as if a similar situation could happen to me	Identification
13	I felt as if I was experiencing the events in the video	Identification
14	I felt as if the events in the video were happening to me	Affective empathy
15	I felt as if I was in the same situation	Affective empathy
16	I felt as if I was in the middle of the situation	Affective empathy
17	I felt as if the characters’ feelings were my own	Affective empathy
18	I felt as if I were one of the characters	Affective empathy
19	I experienced similar feelings as a character	Affective empathy

The self-assessed empathy measure was the result of the mean score of all the questions. All the questions were rated on a five-point Likert scale. We asked about the degree of agreement with each empathy statement, with the lowest scale labeled “strongly disagree” and the highest as “strongly agree.” We also measured subjects’ arousal and valence ratings on the video content, in addition to the ratings on how they felt after viewing the content on a five-point Likert scale.

#### Micromovements signal extraction

The minute head movements, which have been called the ballistocardiography (BCG) have been considered as a method of calculating blood flow from the heart via the carotid arteries^21^. Balakrishnan et al.^35^ confirmed that ballistocardiographic changes are reflected in the face and can be measured at a distance. In this section, we describe how unconscious head movements, which we call micromovements, were elicited from the video data and how the similarity of the micromovement signals was measured (i.e., cross-entropy measure). We used the method established by Lee et al.^38^, as follows: (1) Face Detection, (2) Area Selection, (3) Feature Extraction, (4) Feature Tracking, (5) Signal Filtering.

**Figure 6:**
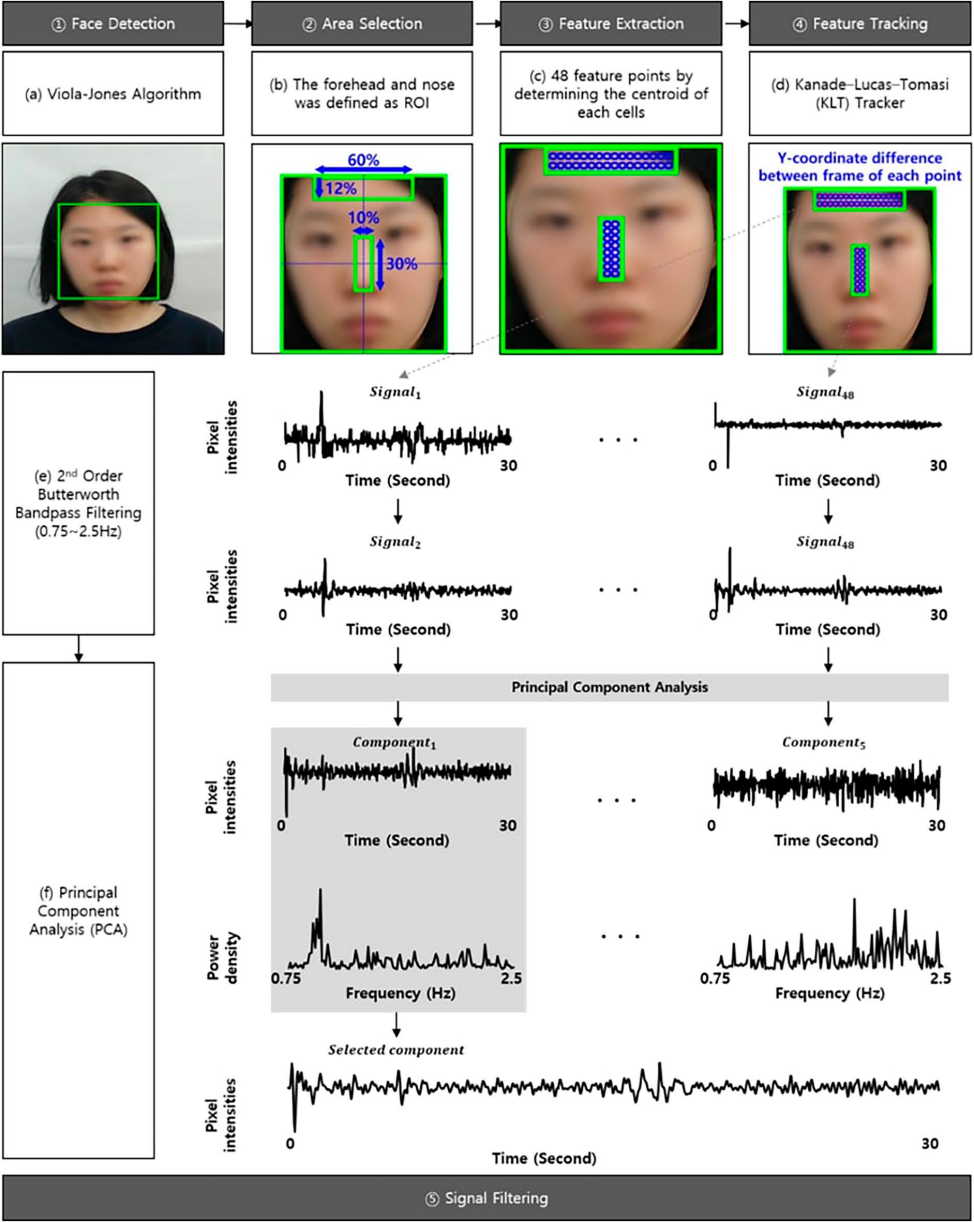
Mean empathy scores of stimuli.

1. *Face Detection* The face was detected from the facial video using the Viola-Jones face detector and was defined as the region of interest (ROI)^61^ as shown (a) in Fig. 6.
2. *Area Selection* As the forehead and nose were more robust to facial expressions than other facial regions, the ROI was categorized into multiple ROIs by cropping the middle 60% of the width and top 12% of the height (i.e., forehead region) and the middle 10% of the width and middle 30% of the height (i.e., nose region) as shown (b) in Fig. 6.
3. *Feature Extraction* Determining the feature point within multiple ROIs is necessary to measure movements induced by BCG changes. The ROIs of the forehead and nose regions were segregated into cells using 16 × 2 and 2 × 8 grids, respectively. We employed 48 feature points by determining the centroid of each cell as a feature point as shown (c) in Fig. 6.
4. *Feature Tracking* The movements were measured by tracking the y-coordination difference between frames of each feature point using the Kanade-Lucas-Tomasi (KLT) tracker because the BCG movements were generated up and down by the heartbeat^62−64^ as shown (d) in Fig. 6.
5. *Signal Filtering* The movements measured from the face are a combination of facial expressions, voluntary head movements, and micromovements. It is essential to eliminate the motion artifacts. First, the movements were filtered using a second-order Butterworth bandpass filter with a cut-off of 0.75–2.5 Hz corresponding to 45–150 bpm as shown (e) in Fig. 6. The movements were then normalized from their mean value (μ) and standard deviation (σ) using the z-score. If the movements exceeded μ ± 2σ, they were determined to be noise due to subtle movements, and their mean value was corrected. Finally, principal component analysis (PCA) was performed to estimate the micromovement from the mixed movements by decomposing the noise from facial expressions and voluntary head movements as shown (f) in Fig. 6. We extracted five components using PCA and then selected one component with the highest peak in their power spectrum converted using a fast Fourier transform (FFT). The selected component was finally determined to be a micromovement.

**Figure 7:**
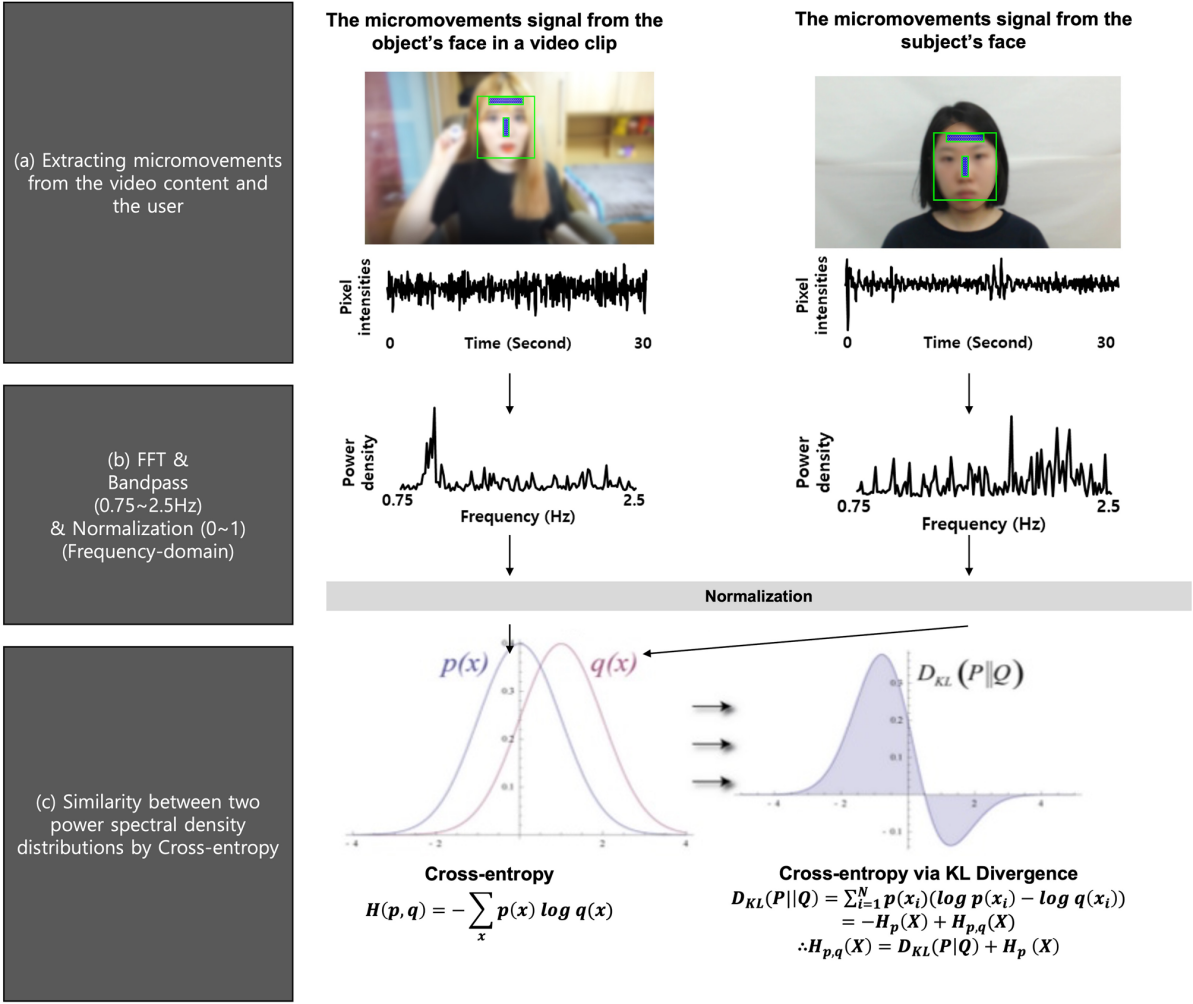
Calculation of cross-entropy between two probability distributions.

Cross-entropy is an unsimilarity metric between two probability distributions. In short, the more the two distributions converge, the closer is the cross-entropy to zero. We hypothesized that higher empathethic response represents lower cross-entropy measure. The cross-entropy measure has been calculated as shown in Fig. 7.

After extracting the micromovements from each subject's face and the object's face (a), The micromovements, which were time-series signals, were converted into the power spectrum signals using fast fourier transformation (FFT) as shown (b) in Fig. 7. The power spectrum signals were considered a normalized probability distribution to calculate cross-entropy measures. The cross-entropy was calculated via KL divergence between these two power spectral density distributions as shown (c) in Fig. 7.

#### Heart rate variability (HRV)

Besides facial data, we recorded ECG readings during the 240-s video presentation. We utilized a 180-s sliding window to assess variations in continuous heart rate data. From this ECG window, HRVs were derived at 1-s intervals. We transformed the participants' ECG time-series data from the 180-s window into frequency bands via an FFT. For reliable short-term ECG readings (under 5 min), we determined the SDNN and RMSSD as time-domain HRV parameters. SDNN evaluates the overall variability of heartbeats, capturing all influential factors during the heartbeat process, both autonomic and others. Conversely, RMSSD assesses the variability between consecutive heartbeats, often linked to the parasympathetic activity within the autonomic nervous system. We also computed the total power by aggregating the power across frequency ranges from 0.04 to 0.4 Hz, providing a holistic measure of heart rate variability, encompassing both low and high-frequency fluctuations. Table 2 outlines the HRV measurements used in this study.

**Table 2:** Heart rate variability variables.

Variable	Unit	Definition
SDNN	msec^2^	Standard deviation of NN Intervals
RMSSD	msec^2^	Root mean square of successive differences
Total power	msec^2^	Total power of the heart rate signal across all frequency components within 0.04 ~ 0.4 Hz frequency range

### Institutional review board statement

The study was conducted according to the guidelines of the Declaration of Helsinki, and approved by the Institutional Review Board of the Sangmyung University, Seoul, Korea (SMUIRB C-2019-015).

## Analysis

We designed videos to elicit varied arousal and valence emotions, as shown in Fig. 5. We were interested in the differential impact of videos on subjects’ self-assessed emotions and self-assessed empathy, physiological responses (i.e., HRV), and cross-entropy measures from the micromovement signals.

We first analyzed self-assessed emotions for each arousal and valence dimension, as described in "Self-assessed emotions" section. We then analyzed HRV to investigate the physiological responses of the emotions in "Physiological response (HRV)" section. While there are several HRV variables, as shown in Table 2, we only reported variables with significant results. We then analyzed self-assessed empathy for each arousal and valence dimension, as described in "Self-assessed empathy" section. The self-assessed empathy was scored by averaging the 19 items in the self-assessed empathy questionnaire.

Finally, we analyzed whether there was a significant difference in the cross-entropy measure in "Cross-entropy measure" section. We hypothesized that the smaller the cross-entropy, the higher the similarity between the micromovement signals. The hypothesis was tested by correlation analysis between the cross-entropy measure and self-assessed empathy. Also, the difference in the cross-entropy measure in emotions was analyzed. In this study, we used Mann–Whitney U tests due to the data distribution not meeting the normality assumption, necessitating non-parametric analysis.

## Results

### Self-assessed emotions

**Figure 8:**
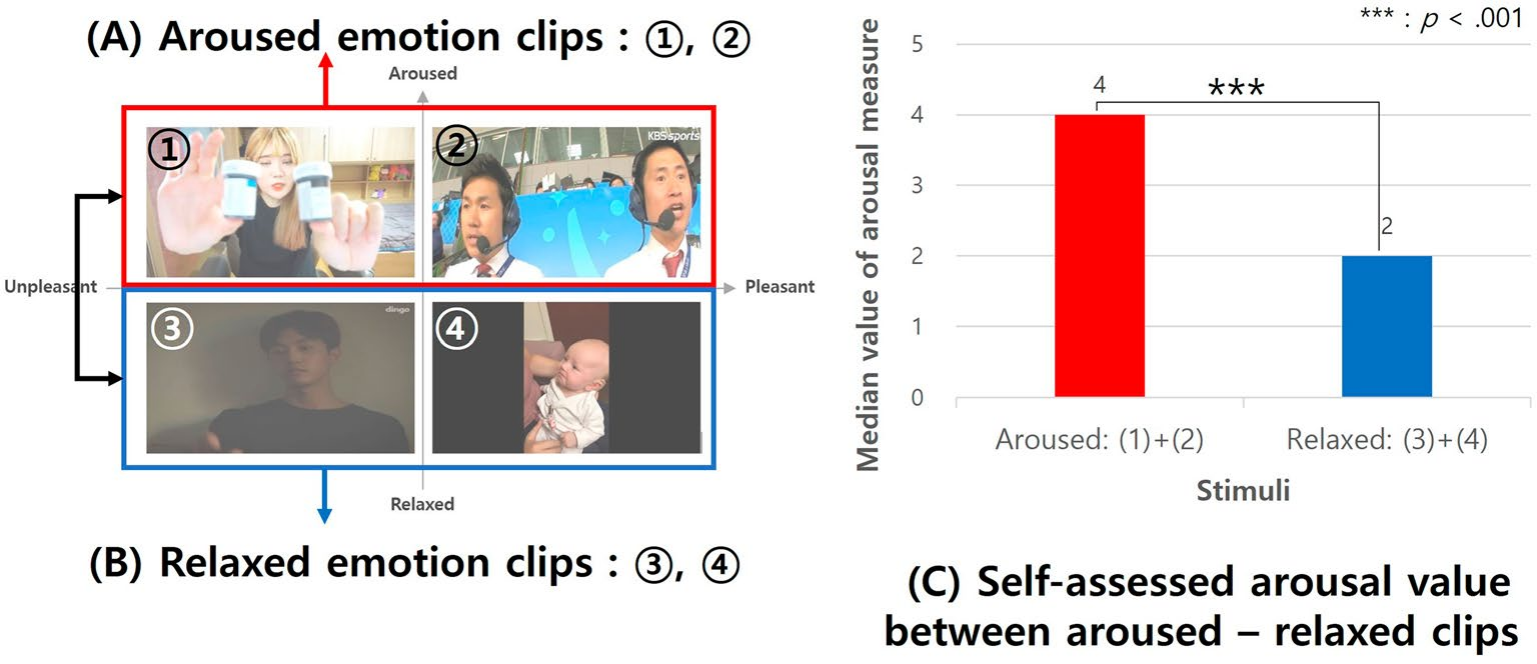
Self-assessed emotions between aroused-relaxed stimuli.

We examined whether the emotional stimuli, which were divided into two categories—Aroused clips (A) and Relaxed clips (B)—elicited the expected arousal levels, as depicted in Fig. 8. Using Mann–Whitney tests, we found a significant difference in the self-assessed arousal scores determined by the median values from the 5-point Likert arousal measure. The scores were notably higher for the Aroused emotion clips (Median = 4) compared to the Relaxed emotion clips (Median = 2), with U = 3717, *p *< 0.001, and Cohen's d =  − 0.055, 95% CI [− 0.069, − 0.041], as illustrated in (C).

**Figure 9:**
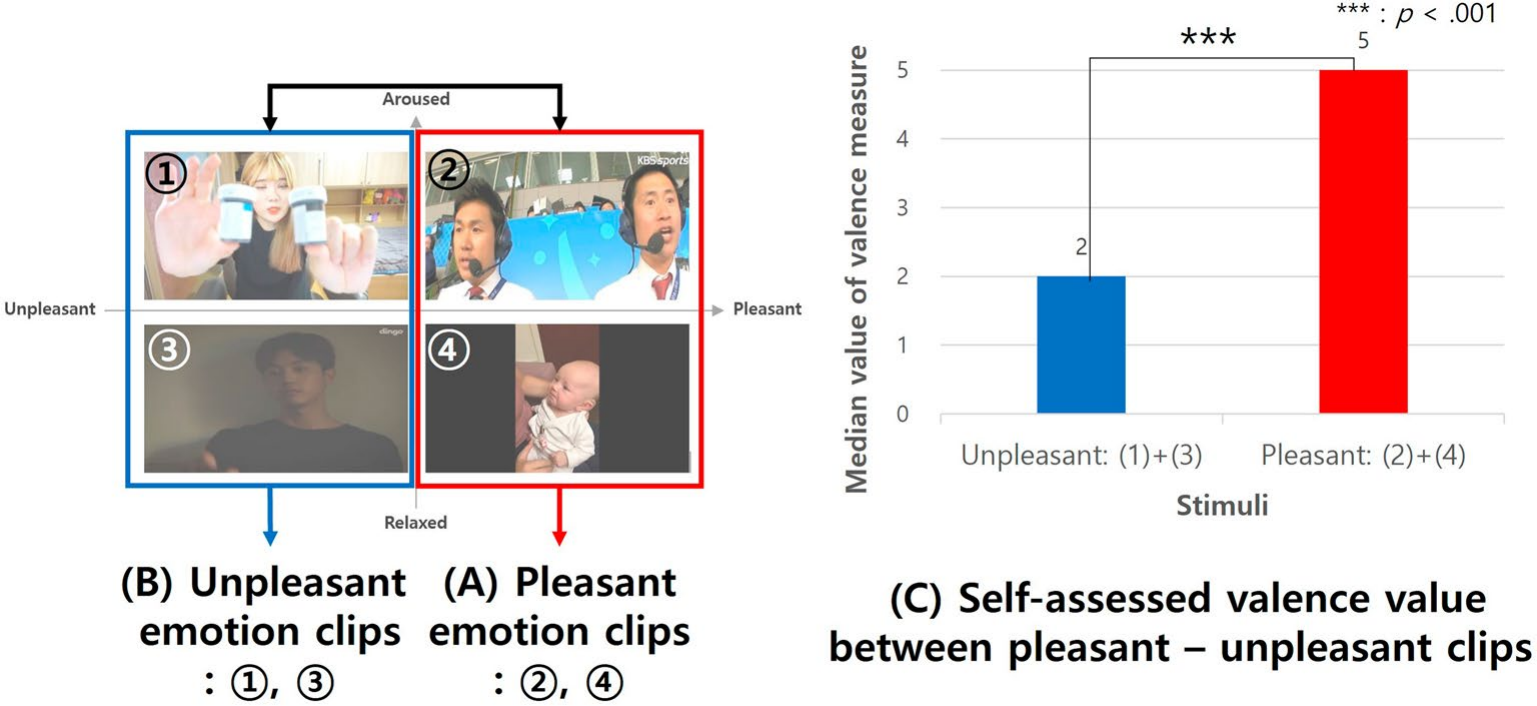
Self-assessed emotions between pleasant-unpleasant stimuli.

We then examined the emotional stimuli, categorized into two groups—Pleasant clips (A) and Unpleasant clips (B)—to determine if they elicited the expected valence levels, as presented in Fig. 9. Using Mann–Whitney tests, we identified a significant difference in the self-assessed valence scores, revealed by the median values from the 5-point Likert valence measure. The scores for Pleasant emotion clips (Median = 5) were significantly higher than those for Unpleasant emotion clips (Median = 2), evidenced by U = 624, *p *< 0.001, Cohen's d =  − 0.106, and a 95% CI of [− 0.133, − 0.079], as detailed in (C).

### Physiological response (HRV)

**Figure 10:**
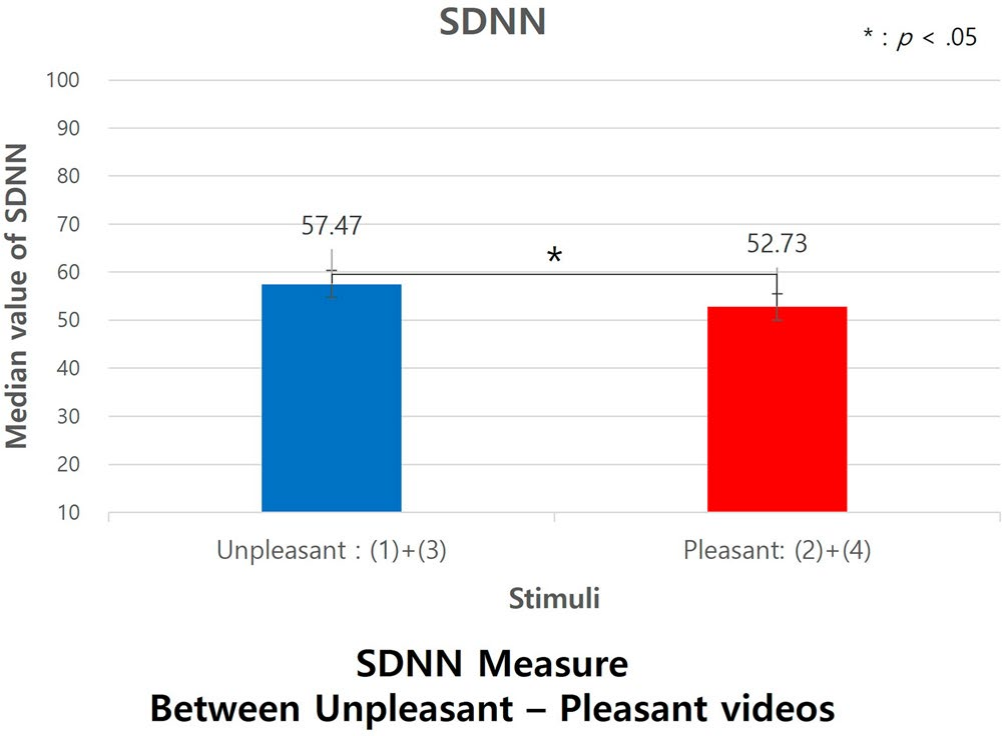
SDNN measure between pleasant-unpleasant stimuli.

We examined the physiological responses, as measured by SDNN and Total power, to the emotional stimuli categorized into two groups, Unpleasant videos and Pleasant videos. Using Mann–Whitney tests, we found a significant difference in the SDNN measure. Specifically, the scores for Unpleasant emotion videos (Median = 57.47) were notably higher than those for the Pleasant emotion videos (Median = 52.73), with U = 6089, *p *= 0.039, Cohen's d =  − 0.1335, and a 95% CI of [− 0.167, − 0.1], as depicted in Fig. 10. The effect size, Cohen's d, suggests a small effect.

**Figure 11:**
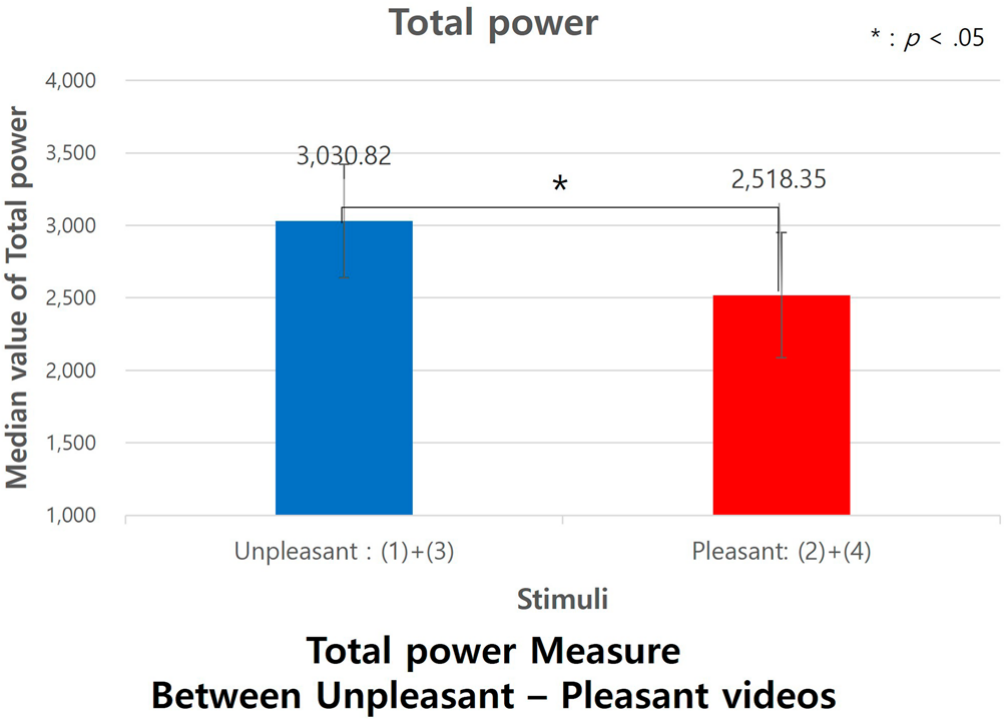
Total power measure between pleasant-unpleasant stimuli.

We examined the Total power measure in response to the emotional stimuli divided into two groups—Unpleasant videos and Pleasant videos. Using Mann–Whitney tests, we identified a significant difference in this measure. Specifically, the values for Unpleasant emotion videos (Median = 3030.82) were significantly higher than for the Pleasant emotion videos (Median = 2518.35), with U = 6066, *p *= 0.035, Cohen's d =  − 0.1335, and a 95% CI of [− 0.167, − 0.1], as presented in Fig. 11. The effect size, Cohen's d, was − 0.136, indicating a small effect. There was no significant variance in HRV measures between the aroused and relaxed video conditions.

### Self-assessed empathy

**Figure 12:**
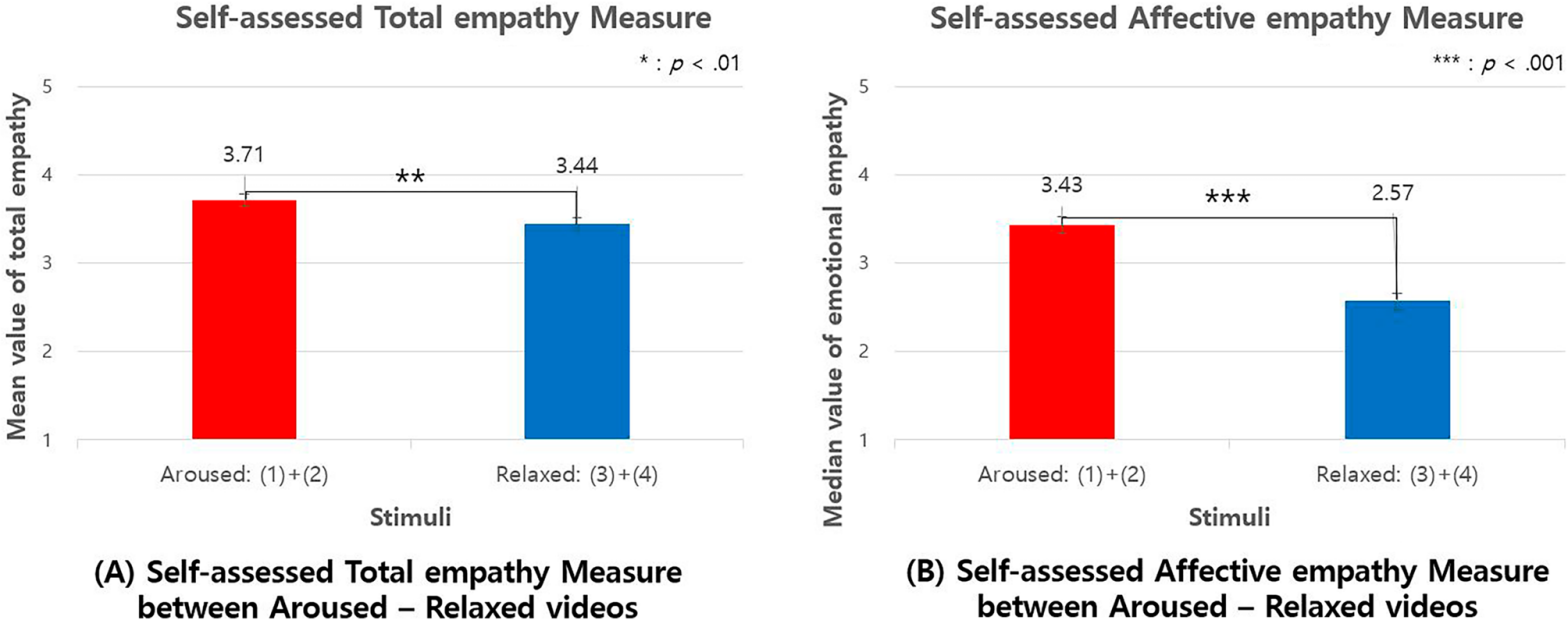
Self-assessed empathy between aroused-relaxed stimuli.

Figure 12 shows a significant difference in self-assessed empathy between the aroused and relaxed emotion conditions (*t*[238] = 2.689, *p *= 0.008, *d* =  − 0.089, 95% CI [− 0.178, 0.038]). The total empathy value (A), which was the average score of 19 self-assessed empathy items, was higher in the aroused condition (M = 3.37, SD = 0.78) than in the relaxed condition (M = 3.44, SD = 0.77). (B) shows a significant difference in the affective empathy items (6 out of 19 items) between the aroused and relaxed emotion conditions. The average scores of affective empathy items were higher in the aroused condition (Median = 3.43) than in the relaxed condition (Median = 2.57), *U* = 4845, *p* < 0.001, Cohen's *d* =  − 0.036, 95% CI [− 0.041, − 0.031]. There was no significant difference in cognitive empathy items or identification items.

**Figure 13:**
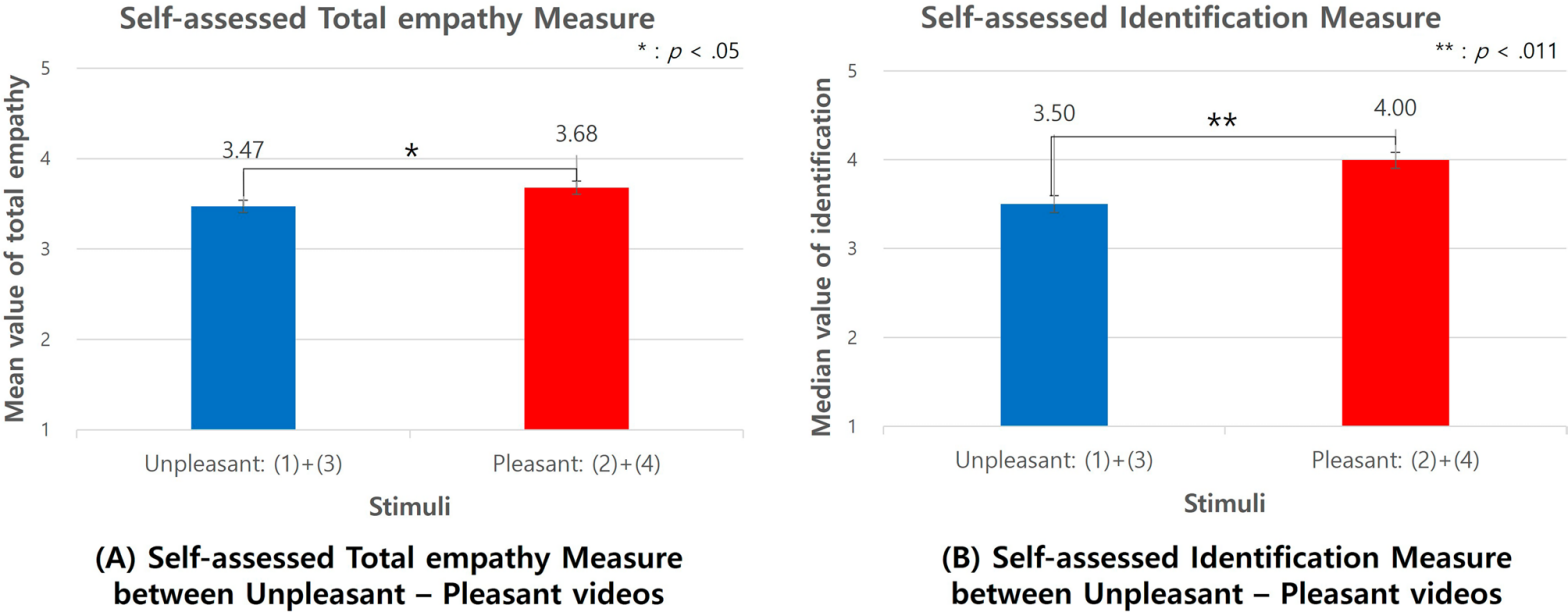
Self-assessed empathy between pleasant-unpleasant stimuli.

As shown in Fig. 13A, there was also a significant difference in self-assessed empathy for valence (*t*[238] = 2.05, *p* = 0.041, *d* = 0.276, 95% CI [0.088, 0.332]). The self-assessed empathy measure was higher in the pleasant emotion (M = 3.68, SD = 0.80) than in the unpleasant emotion (M = 3.47, SD = 0.75). Unlike the arousal condition, there was a significant difference in identification items (7 out of 19 items) (t[238] = 0.252, *p *= 0.004, d = 0.387, 95% CI [0.183, 0.557]). The mean median value of identification score was higher in the pleasant condition (Median = 4.00) (M = 3.80, SD = 0.93) than in the unpleasant (Median = 3.50), U = 5712, *p *= 0.006, Cohen's d = 0.958, 95% CI [− 10560.76, 10,562.68] (M = 3.43, SD = 1.04). Overall, self-assessed empathy measures were higher in aroused and pleasant conditions.

### Cross-entropy measure

**Figure 14:**
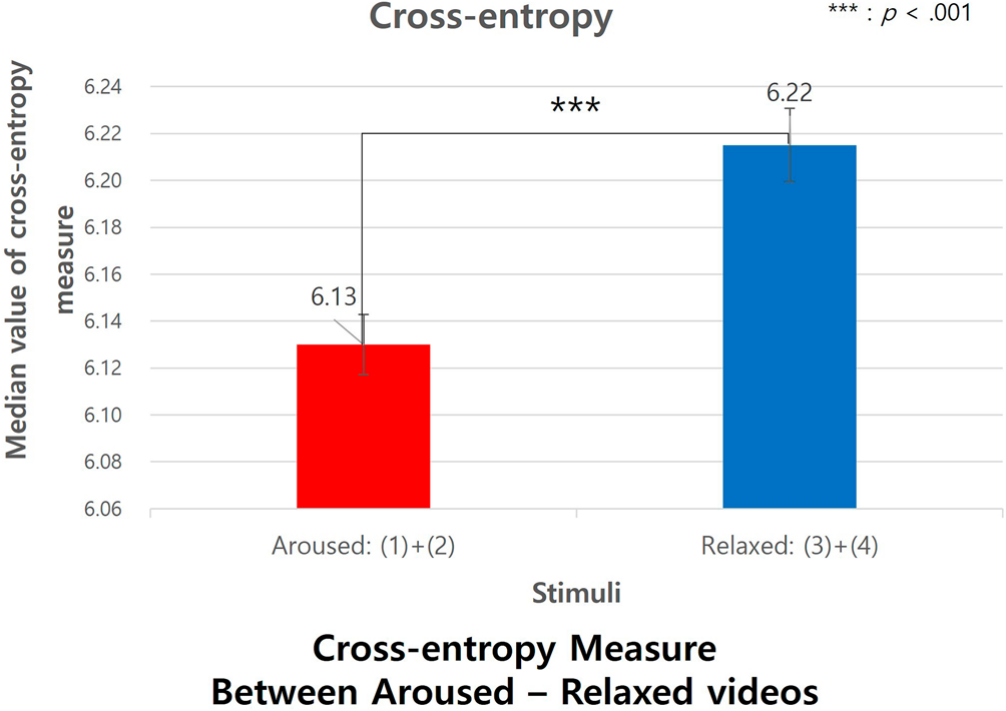
Cross-entropy measure between aroused-relaxed stimuli.

There was a significant difference in the cross-entropy measure between the aroused and the relaxed, as shown in Fig. 14. The cross-entropy, which was the similarity between a subject and an object (i.e., person), was lower in the aroused condition (Median = 6.13) than in the relaxed condition (Median = 6.22), U = 4773, *p *< 0.001, Cohen's d = 0.825, 95% CI [− 35684.71, 35,686.36]. However, there was no significant difference in the cross-entropy measure between the pleasant and unpleasant conditions.

The correlation analysis showed a significant negative correlation between cross-entropy and self-assessed empathy (*r*[238] = − 0.215, *p *= 0.001, *d* = − 0.473, 95% CI [− 0.558, − 0.388]). However, there was a weak correlation (20%), as shown in the r-squared value.

## Discussion

In our study, we pioneered the development and validation of an innovative quantitative measure to assess empathy within videos. To our knowledge, this measure is the first of its kind capable of quantifying empathy for video content without necessitating direct physical contact with the viewer. This was achieved by utilizing a cross-entropy metric which evaluates the depth of viewers' empathy towards the content, as mirrored by the alignment of their micro-movements with those of the characters featured in the video. Notably, we observed a significant difference in measures between the aroused and relaxed conditions, as calculated by cross-entropy between the subject and object in the video.

In summary, the study found that the stimuli used in the experiment successfully induced the intended arousal in the subjects, which was confirmed through both self-reported emotions and heart rate variability (HRV) responses. The HRV measure also suggested that higher arousal was associated with stronger activity in the sympathetic nervous system (SNS). Additionally, the study found that subjects were more empathetic towards the object in the arousal condition compared to the relaxed condition, as indicated by higher self-assessed empathy and lower cross-entropy measure (i.e., stronger emotional contagion).

Conversely, the cross-entropy differences between unpleasant and pleasant states were not significant. However, in HRV measurements, both SDNN and Total power values were higher during the unpleasant state. This is interpreted as the body's response to stress stimuli, suggesting that the variation in the autonomic nervous system appears more active in responding to the stimuli. In summary, subjects exhibited greater empathy in aroused states compared to relaxed states. However, in areas that act as stressors, such as the unpleasant domain, subjective empathy was found to decrease.

Delving into the methodological nuances, cross-entropy, a benchmark for similarity, approaches zero as the resemblance between two signals intensifies. Drawing from Shannon's information theory (1948), the predictability of signals escalates with increased similarity. While there are several similarity metrics available (e.g., cosine similarity, Manhattan, Euclidean distance), we opted for cross-entropy due to its ability to capture the subtle changes that reflect periodic cardiac signals. Moreover, since the cardiac response varies considerably among individuals, distance-based measures such as Euclidean distance, which average positional differences, are not as effective. To assess the overall similarity between the two signal dispositions using a normal distribution, we utilized cross-entropy. The cross-entropy measures showed sufficient sensitivity to detect differences, with values converging towards either zero or one.

We measured the micromovement signals using rBCG, a non-contact measure of the BCG that utilizes a camera. The heart generates ballistic forces that propagate to the head through the carotid artery, resulting in vertical movements of the head and face. Therefore, we extracted the vertical movements and eliminated any noise caused by facial expressions using the denoising method detailed in "Micromovements signal extraction" section. Previous studies^11,14,21,30,65^ have proposed coherence of heart rhythm between two people as a measure of empathy. However, this method has several limitations. It requires the subjects to attach an ECG or PPG sensor to measure their heart responses, making it impractical for situations involving video content and the viewer. To overcome this limitation, we developed a contactless method using rBCG to measure empathy towards video content. We validated that the cross-entropy between micromovement signals could be used as a proxy for coherence of heart rhythms.

In the present study, we utilized self-report measures to assess empathy via structured questionnaires. Notably, while certain items were designed to probe cognitive empathy, this does not conclusively signify an authentic grasp of the video's contextual nuances and situational intricacies; rather, it reflects a participant's subjective perception. In subsequent research, we aim to incorporate a qualitative approach, possibly through post-experiment interviews, to more thoroughly assess the depth of participants' comprehension of the presented content.

Our study also has a notable limitation in that the stimuli are limited to a single person (i.e., actor) within the media. To advance our understanding of empathy measurement, future research may investigate how videos featuring multiple persons or videos without a person could be used. For videos featuring multiple persons, it would be crucial to analyze the representative emotion of the group. Micromovements analysis could determine the social identification of a group in media^66^, particularly in terms of co-movement between individuals. As empathy is a complex construct, future studies should consider constructing multi-regression models that include critical moderating factors such as gender and expressivity.

## Data Availability

The datasets used and analysed during the current study are available from the corresponding author on reasonable request.
